# Melatonin: A Novel Indolamine in Oral Health and Disease

**DOI:** 10.1155/2012/720185

**Published:** 2012-07-31

**Authors:** V. K. Chava, K. Sirisha

**Affiliations:** Department of Periodontics, Narayana Dental College & Hospital, Nellore, Andhra Pradesh, Nellore 524002, India

## Abstract

This paper attempts to summarise the findings accumulated within the last few years concerning the hormone of darkness “melatonin.” Based on its origin, from the pineal gland until recently it was portrayed exclusively as a hormone. Due to its lipophilic nature, it is accessible to every cell. Thus, in the classic sense it is a cell protector rather than a hormone. Recent studies, by Claustrat et al. (2005), detected few extrapineal sources of melatonin like retina, gastrointestinal tract, and salivary glands. Due to these sources, research by Cutando et al. (2007), is trying to explore the implications of melatonin in the oral cavity, in addition to its physiologic anti-oxidant, immunomodulatory and oncostatic functions at systemic level that may be receptor dependent or independent. Recently, certain in vivo studies by Shimozuma et al. (2011), detected the secretion of melatonin from salivary glands further emphasising its local activity. Thus, within our confines the effects of melatonin in the mouth are reviewed, adding a note on therapeutic potentials of melatonin both systemically and orally.

## 1. Introduction

In light of the growing evidence, researchers are attempting to understand how the body naturally “turns off” inflammation. Melatonin (N-acetyl-5-methoxytryptamine) is one such powerful hormone derived from an essential amino acid tryptophan [1]. It has various local and systemic functions playing a critical role in controlling inflammatory reactions. It is predominantly synthesised and secreted from pineal gland and other extrapineal sources like retina, gastrointestinal tract, lens, and immune system cells [2]. The effects of melatonin were described in 1917 but were first isolated from bovine pineal gland and structurally identified in 1958 by Lerner and colleagues [3]. Melatonin derives its name from serotonin, based on its ability to blanch the skin of amphibians [4]. The unique, highly lipophilic nature of melatonin makes it accessible to every cell. It is found in high concentrations in the bone marrow, intestine, and at the sub-cellular level in the mitochondria and nucleus [5]. Recently, its presence is detected in saliva and gingival crevicular fluid. Melatonin is said to play a significant role in protecting the oral cavity through its antioxidant, immunomodulatory, oncostatic, and other functions [6]. Based on this evolving evidence, an attempt is made to review melatonin's synthesis, effects and proposed therapeutic potentials.

## 2. Synthesis

**Figure 1:**
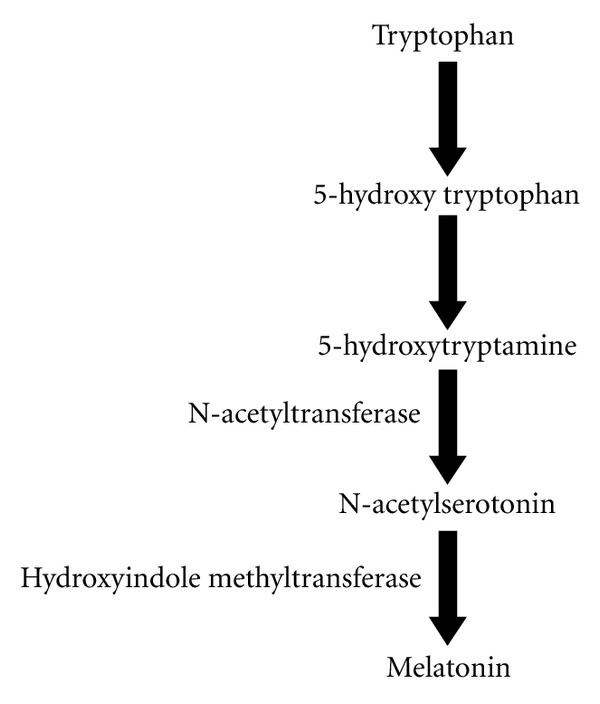
Biochemical steps for melatonin synthesis in pinealocyte.

Pinealocytes are responsible for melatonin production through a series of well-known reactions [7, 8]. It requires polysynaptic activation of beta-adrenergic receptors, which are indirectly regulated by neural stimulus from suprachiasmatic nucleus (SCN). Information on light/dark environments is transmitted via the retinohypothalamic tract to the suprachiasmatic nucleus. Thereafter an electrical neural signal is transferred to the upper thoracic cord and superior cervical ganglia after which it is conveyed from the postganglionic sympathetic fibres to the pineal gland [9]. These postsynaptic terminals release norepinephrine which activates adrenergic receptors in the pinealocyte membrane. Pinealocytes take up tryptophan from the blood followed by the enzymatic reactions as shown in Figure 1, contributing to the synthesis of melatonin with the rate limiting enzyme being N-acetyltransferase (AANAT). Once produced, melatonin is quickly discharged into the capillary bed in the pineal gland and possibly directly into cerebrospinal fluid of third ventricle [10].

## 3. Melatonin Receptors

Melatonin has specific membrane and nuclear receptors that have been cloned and three subtypes have been identified [11, 12]. They were initially named Mel-1a, and Mel-1b, Mel-1c [13]. The Mel-1a receptor gene has been mapped to human chromosome 4q35.1. Its primary expression is in the pars tuberalis of the pituitary gland and suprachiasmatic nucleus. Mel-1b has been mapped to chromosome 11q21-22, and its main expression is in the retina and brain. Mel-1c is not found in mammals. Mel-1a, Mel-1b are now renamed as MT1 and MT2 by International Union of Basic and Clinical Pharmacology (IUPHAR) [14]. These two are members of a group of membrane receptors known as G-protein-coupled receptors that share a large part of amino acid sequences. The recently discovered MT3 receptor is a cytosolic enzyme, quinone reductase. Using gene knockout technology results to date suggests that the phase-shifting receptor is MT2, while MT1 is associated with acute suppression of suprachiasmatic nucleus electrical activity in addition to its important actions within the pars tuberalis; MT1 potentiates adrenergic vasoconstriction and MT2 modulates dopamine release in the retina [15–17].

## 4. Circadian Rhythm

The rhythmic production of melatonin is a consequence of neural impulses from the biologic clock, that is, from SCN and hypothalamus [1, 18]. It is known as the “chemical expression of darkness” as most of its synthesis occurs during night [19]. In healthy individuals, peak serum melatonin levels are seen between 12.00 a.m–2.00 a.m. and 2.00–4.00 a.m, with minimum secretion occurring during the day 12.00 p.m–2.00 p.m [20]. Following its secretion unbound melatonin diffuses passively into the saliva and oral mucosa to enter the oral cavity [21]. So, salivary melatonin represents the percentage of free melatonin [22]. The presence of melatonin in saliva is confirmed by several techniques such as automated solid phase extraction, high-performance liquid chromatography, and fluorescence detection [23]. Salivary melatonin levels (2–4 pg/mL) [24] form 24–33% of the plasma melatonin levels [25]. Factors such as smoking, exposure to light, alcohol consumption, and aging lower the levels of salivary melatonin with no variability in terms of gender [26]. Recent evidence has observed its presence in the gingival crevicular fluid (GCF). GCF melatonin levels are 60% lower than serum levels and 30% lower than salivary levels [6]. Measurement of salivary melatonin is a reliable technique to monitor the circadian rhythms of melatonin.

## 5. Physiological Functions

Since its discovery, melatonin has shown to have a variety of important functions in all species [27]. These systemic and oral functions are indeed related to its hormonal properties as shown in Table 1.

### 5.1. Effects in Oral Cavity

Current research portrays melatonin as a cell protector rather than a hormone, due to its presence in extra-pineal organs including oral cavity [28]. It passively diffuses into the oral cavity and is released into the saliva [29]. A significant correlation between concentrations of melatonin in saliva and serum was reported with a general conclusion that melatonin concentration is a reliable index of serum melatonin levels [30].

## 6. Role of Melatonin in Oral Cancerous Lesions

 Melatonin plays a pivotal role in many inflammatory processes of oral cavity and thus is effective in treating pathologies [31] like squamous cell carcinoma and epidermoid carcinoma. In relation to oral cancer, it is speculated that exogenous restoration of melatonin receptor 1a inhibited the growth of oral squamous cell carcinoma cells, lacking its expression [32]. By its actions against ROS, melatonin may protect against precancerous oral diseases like leukoplakia and lichen planus [32, 33]. Melatonin also counteracts the negative effects of immunosuppressive drug therapy by acting on T-helper lymphocytes, lymphokines such as gamma interferon and IL-2 [34].

## 7. Role of Melatonin in Tooth Development

 Melatonin may play a role in physiologic tooth development and growth by regulation of odontogenic cells in tooth germs [35]. Immunohistochemical analysis revealed that melatonin 1a receptor (Me 1a R) was expressed in secretory ameloblasts, the cells of stratum intermedium and stellate reticulum, external dental epithelial cells, odontoblasts, and dental sac cells. Seasonal variation in the release of melatonin was associated with the changes in development of caries in hamsters [36].

## 8. Role of Melatonin in Inflammatory Conditions of the Oral Cavity

 GCF melatonin levels play a significant role in periodontal disease. The pathogenesis of periodontitis is related to increased oxidative stress, which in turn leads to tissue damage and bone loss. The antioxidative and free radical scavenging action of melatonin may reduce this tissue damage [37]. In addition, the positive effects of melatonin and its derivatives on inflammatory mediators and bone cells may be beneficial in improving periodontal health. Recent evidence [37] proved that salivary melatonin levels may vary according to the degree of periodontal disease. A negative association was found between salivary melatonin levels and periodontal disease severity. Consequently, decreased saliva and melatonin production with age predisposes older individuals to increased risk of oral and periodontal diseases. Dental procedures including tooth extraction may result in local inflammation and oxidative stress in the oral cavity. Upon local administration, the antioxidant action of melatonin may be useful in counteracting this oxidative stress [38, 39].

## 9. Role of Melatonin in Osseointegration and Regeneration

 Melatonin was reported to stimulate the growth of new bone around implants in the tibia of rats [38]. It has biologic significance in terms of osseointegration around implants. 2 weeks after implant insertion, melatonin significantly increased all parameters of osseointegration like bone to implant contact (BIC), total peri-implant bone, interthread bone, and new bone formation. In general, implants impregnated with topical melatonin at the time of placement showed more trabecular bone and higher trabecular density at implant contact [40, 41]. Osseointegration was reported to be more effective when melatonin was mixed with collagenised porcine bone [42, 43]. Melatonin at micro-molecular concentration promotes the proliferation of human mandibular cells (HOB-M) and cells of a human osteoblastic cellular line (SV-HFO). This effect is dose dependent and is maximum at concentrations of 50 micrometers [44]. At concentrations ranging from 5 to 500 micrometers, melatonin lowers the expression of mRNA from the RANK and increases levels of both OPG as well as mRNA from the OPG in preosteoblast cell lines [45]. Due to these actions on bone, its use as a biomimetic agent during endosseous dental implant surgery is proposed [46].

## 10. Role in Reducing Toxicity due to Dental Materials

 Several cytotoxic and genotoxic effects of dental methacrylate monomers promote oxidative processes. Melatonin's antioxidative action may protect against these effects by reducing oxidative DNA damage induced by methacrylates [47]. Melatonin when used as a component of dental materials exhibited biocompatibility without altering the properties of these dental materials. Alternatively, the regular use of melatonin oral rinse may reduce the side effects of methacrylate monomers [47].

## 11. Role in Modification of Salivary Components

 Novel evidence proposed that melatonin induced protein synthesis in the rat parotid gland and thereby affects glandular activity. This effect is MT-1- and MT-2-receptor-mediated and is primarily dependent on nitric oxide generation via the activity of neuronal-type nitric oxide synthase. This enzyme probably originated from parenchymal cells of the parotid gland. This novel action of melatonin may increase its clinical implications in the treatment of xerostomia, caries, periodontitis, oral mucosal infections, salivary gland inflammation, and wound healing [48]. Summary of the Effects of melatonin in the oral cavity is shown in (Table 2).

## 12. New Sources of Melatonin

 The previously mentioned finding put forth the quest for new sources of melatonin in the oral cavity. They evaluated the expression of melatonin-synthesising enzyme aryl-alkyl-amine N-acetyl transferase (AANAT) and hydroxyl indole-O-methyl transferase (HIONT) mRNA in rat submandibular gland by quantitative reverse transcription polymerase chain reaction. They observed expression of AANAT in the epithelial cells of striated ducts in rat salivary glands and expression of AANAT, HIONT, and melatonin in epithelial cells of striated ducts in human submandibular glands. In addition, the expression of most potent melatonin receptor (melatonin 1a) in rat buccal mucosa was confirmed [49]. Melatonin at physiologic concentrations increased nerve growth factor synthesis in mouse submandibular gland [50]. So, these oral sources of melatonin may alter salivary melatonin levels and thereby promote better oral health. So, without doubt all these actions have important consequences at the time of treatment of our high-risk dental patients who have in one way or the other an altered immunological system.

## 13. Therapeutic Potential of Melatonin

 The antioxidant and free radical scavenging properties of melatonin might justify its therapeutic use in the treatment of disease like Parkinson's disease [51], Alzheimer's disease [52], epilepsy [53], infections, and inflammatory disorders [54]. Melatonin is a known antineoplastic agent used in the treatment of cancers of lungs, kidney, prostate gland, stomach, and intestine [55, 56]. Melatonin can also be used in the palliative treatment of cancer due to its anticachetic, antiasthecnic, and thrombopoietic properties [57]. It is a proven powerful cytostatic drug in vitro as well as in vivo. It induces apoptosis in human neuroblastoma cells and inhibits cell growth of glioma cells [55, 58, 59]. A favourable effect of local application of melatonin in the alveolar sockets following molar and premolar extractions was seen in beagle dogs [38]. Melatonin protects the oral cavity and gastrointestinal tract from conditions such as stomatitis, oesophagitis, and peptic ulcer [22]. Melatonin is a novel oesophageal protector acting through COX/Prostaglandin and NOS/NO systems and via activation of sensory nerves [60]. Novel evidence implicates that melatonin may have potential to treat xerostomia by evoking protein/amylase secretion from the parotid gland of anaesthetised rats [61]. In hamsters, the quantity of melatonin consumed in foodstuffs may influence caries incidence [36]. Caries incidence is also found to be less common in winter and autumn when melatonin levels are maximum [62]. Oral melatonin is widely used to reduce the subjective symptoms of jet lag [63]. Melatonin was also found to be useful as a sedative, hypnotic, and anxiolytic. Its use as an oral sedative in dentistry is now being proposed [64].

## 14. Therapeutic Protocols Proposed for Melatonin

 Beneficial antioxidant effects of low doses of melatonin (10 mg/day) are shown in several chronic diseases such as rheumatoid arthritis [65], primary essential hypertension in elderly patients (5 mg/day) [66], type 2 diabetes in elderly patients (5 mg/day) [67], and females suffering from infertility (3 mg/day) [68]. A recent study reported beneficial effects of high doses of melatonin (20 mg/kg) for inhibiting apoptosis and liver damage resulting from oxidative stress in malaria, which could be a novel approach in the treatment of this disease [69]. A formulation containing 2.5 mg melatonin and 100 mg SB-73 (a mixture of magnesium phosphate, fatty acids, and protein extracted from aspergillus oryzae) promoted regression of symptoms of herpes virus infection [70]. The effect of melatonin on the ingestion and destruction of *C. albicans* by the ring dove (*streptopelia risoria*) at different durations of incubation (30–60 mins) with physiological (50 pgmL^−1^ diurnal and 300 pgmL^−1^ nocturnal) as well as with a pharmacological concentration 100 *μ*m of melatonin showed a dose-dependent effect with decrease in superoxide anion levels after incubation [71].

## 15. Negative Effects of Melatonin

Melatonin is a potent adjunctive agent in the treatment of cancer and immune deficiency. However, poorly timed administration can produce opposite effects. Melatonin injections given in the morning stimulate tumour growth, whereas same doses given in the mid-afternoon have no effect but in the evening have a retarding effect [72, 73]. Melatonin administration may unduly prolong the nocturnal melatonin rise or that is given throughout the day may exacerbate bipolar and classic depression [74, 75]. Some people with depression may suffer from a low-melatonin syndrome [76]. Animal studies have shown that moderately large doses of melatonin (equivalent to 30 mg in adult humans) increase light induced damage to retinal photoreceptors [77]. Melatonin caused atherosclerosis in the aorta in hypercholesterolemic rats by suppressing LDL-receptor metabolic pathways [78]. Preliminary animal studies suggest that melatonin may accelerate the development of autoimmune conditions [79]. 

## 16. Conclusion

 Traditionally, melatonin was considered as the principal secretory hormone of pineal gland. Its profound systemic effects as a cell protector stimulated the quest for other extrapineal sources and functions. Novel evidence brought to light that this chemical of darkness has oral sources and implications. Based on this evidence, this paper attempts to concise the role of melatonin in oral health and disease mentioning a note on its therapeutic potential. Further studies need to focus their attention on therapeutic uses of melatonin as a coadjuvant in oral hygiene aids and as an antimicrobial in local therapy to promote it as a natural inhibitor of inflammation.

## Figures and Tables

**Table 1:** Properties and functions of melatonin.

Functions	Mechanism of action	Authors and date of publication
Anti-oxidant	Direct effects: neutralizes a variety of ROS like OH, ROO, H_2_O_2_, and O_2_ (nonreceptor mediated). Interacts with the lipid bilayers and stabilizes mitochondrial membranes thereby improving electron transport chain. Indirect effects: (i) regulates nitric oxide production by interaction with nitric oxide synthesis, (ii) increases gene expression and enzyme activities of glutathione promoting removal of H_2_O_2_ and super oxide dismutase (receptor mediated).	Ganguly et al., 2006 [80], Reiter et al., 1995 [81] Tan et al., 2000 [82], Cagnoli et al., 1995 [83] Bongiorno et al., 2005 [84], Aydogan et al., 2006 [85]

Anti-inflammatory	Inhibits inflammatory enzyme COX-2 by binding to the active sites of COX-1 and COX-2.	Antolín et al., 1996 [86], Rodriguez et al., 2004 [87], Tomás-Zapico and Coto-Montes, 2005 [88]

Immunomodulatory function	Promotes endogenic production of IL-2 concomitant with serum melatonin concentration. Activates CD+4 lymphocytes by increasing the production of IL-2 and IFN-*γ*. Modulates immune functions by activating ng on CD+4 cells and monocytes.	De la rocha et al., 2007 [89] Guerrero et al., 2000 [90], Garcia-Mauriño et al., 1997 [91]

Anticancer effect	Stimulates production of IL-2, thereby stimulates the activity of natural killer cells which intervene in the cytolytic mechanisms. Amplifies the antitumour activity of IL-2. Scavenges reactive oxygen species which a secondary messengers in the signaling pathway leading to cell division. Inhibited the growth of oral squamous cell carcinoma cells. Involved in the pathogenesis of oral precancerous lesions like lichen planus and leukoplakia by free radical scavenging action.	Garcia-Maurino et al., 2000 [92], Cutando et al., 2004 [93], Martin et al., 2006 [59], Lardone et al., 2006 [94], Reiter et al., 2007 [95], Reiter, 2004 [96]

Antiviral effect	Induces production of IL-1*β* which is useful in treating viral infections.	Chaiyarit et al., 2005 [32]

Antimycotic effect	Enhances phagocytic function and reduces oxidative stress originating during candidiasis.	Nunes and Pereira, 2008 [70]

Bone remodelling	Promotes osteoblast differentiation and bone formation. Stimulates synthesis of type 1 collagen fibres in human osteoblasts in vitro. Inhibits bone resorption by interfering with the activity of osteoclasts through its indirect antioxidant and direct free radical scavenging action. Increase genic expression of bone sialoprotein and other protein markers thereby reducing osteoblast differentiation time. Downregulates RANKL-mediated osteoclast formation and activation.	Terron et al., 2002, 2003, 2004 [71, 97, 98], Roth et al., 1999 [99], Nakade et al., 1999 [44], Koyama et al., 2002 [45], Nakade et al., 1999 [44], Yasuda, 2006 [100], Boyce et al., 2006 [101]

**Table 2:** Effects of melatonin in oral cavity.

Effects in the oral cavity	Author & date
Melatonin at physiologic concentrations increases nerve growth factor synthesis in mouse submandibular gland.	Pongsa-Asawapaiboon et al., 1998 [50]
Melatonin promotes proliferation of human mandibular cells (HOB-M) and cells of human osteoblastic cellular line (SV-HFO) in a dose-dependent manner.	Nakade et al., 1999 [44]
Reduces periodontal tissue damage by its antioxidant and free radical scavenging actions on inflammatory mediators and bone cells.	Cutando et al., 2006 [37]
Counteracts oxidative stress induced during tooth extraction.	Cutando et al., 2007 [38, 39]
Melatonin significantly increases all the parameters of osseointegration of implants when topically impregnated at the time of placement. This effect is enhanced when mixed with collagenised bone.	Cutando et al., 2008 [43] Calvo-guirado et al., 2009 [41] Calvo-guirado et al., 2010 [42] Tresguerres et al., 2012 [40]
Regulates odontogenic cells in tooth germs by expression of melatonin 1a receptor in cells promoting tooth development.	Kumasaka et al., 2010 [35]
Melatonin reduces the oxidative DNA damage induced by dental methacrylate monomers.	Blasiak et al., 2011 [47]
Melatonin induces protein synthesis in the rat parotid gland and thereby effects glandular activity in a MT1 and MT2 receptor-mediated mechanism.	Cevik-Aras et al., 2011 [48]
Melatonin inhibits Prevotella intermedia lipopolysaccharide-induced production of nitric oxide and IL-6 in murine macrophages by suppressing NF-*κβ* and STAT1 activity.	Eun-young Choietal, 2011 [102]
